# The effects of injectable trace mineral solutions on beef cattle performance and health during preconditioning and feedlot receiving: a systematic review and meta-analysis

**DOI:** 10.1093/tas/txaf162

**Published:** 2025-12-13

**Authors:** Madeline G McKnight, Kelsey M Harvey, Juliana Ranches, Brandi B Karisch, W Isaac Jumper

**Affiliations:** Department of Animal and Dairy Sciences, Mississippi State University, Starkville, MS, 39762, United States; Department of Animal and Dairy Sciences, Mississippi State University, Starkville, MS, 39762, United States; Prairie Research Unit, Mississippi State University, Prairie, MS, 39756, United States; Eastern Oregon Agricultural Research Center, Oregon State University, Burns, OR, 97720, United States; Department of Animal and Dairy Sciences, Mississippi State University, Starkville, MS, 39762, United States; Department of Pathobiology and Population Medicine, College of Veterinary Medicine Mississippi State University, Starkville, MS, 39762, United States

**Keywords:** average daily gain, beef cattle, injectable trace minerals, meta-analysis, morbidity

## Abstract

During the preconditioning and feedlot receiving phases of the beef cattle industry, animals undergo a myriad of stressors which may lead to decreased animal performance and immune system function. However, the supplementation of various trace minerals may aid in the mitigation of the negative effects attributed to these stressors. Since many animals experience decreased feed intake during these periods, producers may opt to utilize an injectable trace mineral (**ITM**) solution to provide prompt supplementation. However, research surrounding cattle health and production effects of such products is variable. Therefore, a meta-analysis further analyzing the effects of ITM solutions was conducted. Studies were collected through independent literature searches, resulting in 16 studies. Data was extracted as treatment means, a value with the ability to calculate standard deviations, *p*-values, and odds ratios, which were then analyzed within the meta package of R (v. 4.3.2). Overall, the usage of an ITM solution did not affect average daily gain (**ADG**  *P = *0.21) or overall morbidity (*P = *0.20) when compared to a saline injection. Additionally, ADG differed between risk classifications (*P = *0.03) with the usage of an ITM product increasing ADG by 0.12 kg/d in high-risk cattle (no known management history; *P = *0.02) with no effect in low-risk cattle (known management history; *P = *0.93) when compared to a saline injection. Furthermore, the administration of an ITM did not affect ADG within the preconditioning (beginning of preconditioning period; *P = *0.39) but tended to increase ADG (*P = *0.09) by 0.06 kg/day within the feedlot receiving (arrival to feedlot facility) subclasses when compared to a saline injection. Average daily gain did not differ based on the inclusion (*P = *0.49) or exclusion (*P = *0.37) of additional oral supplementation. Additionally, ADG did not differ (*P = *0.15) based on study length, accordingly, ITM usage had no effect in short (≤ 30 days; *P = *0.41) or extended (≥ 60 days; *P = *0.74) studies, but increased ADG by 0.07 kg/d in moderate (30–60 days; *P = *0.04) studies. Lastly, ITM administration did not affect ADG within *B. indicus* (*P = *0.42), but increased ADG (*P = *0.05) by 0.03 kg/d within *B. taurus* cattle and increased ADG (*P = *0.21) by 0.09 kg/d within crossbred cattle. In conclusion, ITM administration did not affect overall ADG or morbidity, but may increase ADG within specific production or animal circumstances.

## Teaser

Injectable trace mineral solutions may be a viable way to provide beef cattle with efficient trace mineral supplementation during periods of increased stress and decreased feed intake, such as during preconditioning and feedlot receiving. This systematic review and meta-analysis evaluates the effects of such solutions on cattle performance and health during these periods.

## Introduction

In order to mitigate the effects of stressors, such as transportation, weaning, and comingling (Cooke 2017), within the preconditioning and feedlot receiving phases of the beef cattle industry, producers may opt to utilize targeted nutritional strategies, including the supplementation of minerals with major focus on trace minerals such as of copper (Cu), manganese (Mn), selenium (Se), and zinc (Zn) due to their role within metabolism and immune system function (Suttle 2021). However, due to decreased feed intake during the commencement of these phases, traditional supplementation methods may not be effective in ensuring animals are meeting trace mineral requirements for maintenance and growth. Additionally, this combined effect of physiological stress and decreased feed intake may lead to increased susceptibility to diseases, such as bovine respiratory disease (**BRD**). Various management strategies, such as metaphylactic antibiotic usage, can be utilized to decrease the incidence of BRD within the preconditioning and feedlot receiving phases, however, growing pressure to reduce antimicrobial use has increased the demand for non-pharmaceutical interventions which can enhance resilience to physiological stress and disease.

An injectable trace mineral (**ITM**) solution containing Cu, Mn, Se, and Zn administered as a single-dose supplementation method during processing may be a potential solution to overcome the negative effects of these stressors and immunosuppression while ensuring animals meet trace mineral requirements. However, research surrounding the effects of ITM products on beef cattle growth performance and health outcomes is variable.

Due to the variability present within current literature, a comprehensive systematic review and meta-analysis surrounding the effects ITM administration to beef cattle during preconditioning or feedlot receiving on growth performance and health outcomes is warranted. Therefore, the objectives of this meta-analysis were to critically evaluate experiments which utilized ITM solutions on performance and health of stressed cattle. Additionally, we aimed to differentiate these outcomes according to animal risk classification, phase receiving supplementation, the usage of additional oral trace mineral supplementation, length of study, and animal breed. We hypothesized that administering a single-dose ITM solution to beef cattle at the onset of preconditioning or feedlot receiving phases will increase growth performance and decrease incidence of overall morbidity compared to cattle not receiving an ITM solution.

## Materials and methods

Published studies utilizing an ITM were compiled and critically reviewed to quantify the effects of ITM administration on beef cattle during the preconditioning and feedlot receiving phases. The primary outcomes of interest for this meta-analysis were cattle average daily gain (**ADG**) and overall morbidity.

### Literature search

A systematic literature search was conducted independently by two authors of the present study utilizing Scopus, EBSCO (Academic Search Premier, Agricola, and Environment Complete), and PubMed as reported in McKnight et al. (2025). The search included terms relevant to the population and intervention outlined in the study objectives such as beef cattle, feedlot, preconditioning, and trace minerals. All literature written in English, or with an available English translation, published from January 1, 2000 to May 31, 2025 was considered, yielding 710 articles. After the removal of duplicates, title and abstract review, and reviewing studies for eligibility based on study population, treatment, and study outcomes available, 16 studies from 12 published manuscripts remained (Table 1).

**Table 1.** A summation of studies including study title, phase, length of study, ITM product, risk classification, the usage of additional trace mineral supplementation, and animal breed.

Study	Phase	Length of study^a^	Risk Classification^b^	Additional Mineral Supplementation^c^	Breed
Arthington et al. (2014) (Exp. 2)	Receiving	Short	Low	N	Crossbred
Caramalac et al. (2021)	Preconditioning	Short	Low	N	*B. taurus*
Clark et al. (2006)	Receiving	Short	Combined	Y	Crossbred
da Silva Zornitta et al. (2021)	Preconditioning	Moderate	Low	N	*B. indicus*
Genther-Schroeder & Hansen (2015) (Study 1)	Receiving	Extended	Low	Y	Crossbred
Genther-Schroeder & Hansen (2015) (Study 2)	Receiving	Extended	Low	Y	Crossbred
Grossi et al. (2025)	Receiving	Moderate	High	N	*B. taurus*
Niedermayer et al. (2017)	Receiving	Moderate	Low	Y	*B. taurus*
Rauch et al. (2018) (Study 1)	Receiving	Moderate	Low	Y	Crossbred
Rauch et al. (2018) (Study 2)	Receiving	Moderate	Low	Y	Crossbred
Richeson and Kegley (2011) (Study 1)	Receiving	Moderate	High	Y	Crossbred
Richeson and Kegley (2011) (Study 2)	Receiving	Moderate	High	Y	Crossbred
Roberts et al. (2016)	Receiving	Moderate	High	N	Crossbred
Vedovatto et al. (2019)	Preconditioning	Extended	Low	Y	*B. indicus*
Vedovatto et al. (2024) (Study 1)	Preconditioning	Extended	Low	Y	*B. indicus*
Vedovatto et al. (2024) (Study 2)	Preconditioning	Extended	Low	Y	*B. indicus*

a Short = <30 days, Moderate = 30–60 days, Extended = >60 days.

b Low = known management or vaccination history, High = no known management or vaccination history.

c Y = Included additional trace mineral supplementation, N = did not include additional trace mineral supplementation.

Studies were included in this meta-analysis if they reported the following: the phase the study was conducted (ie, preconditioning or feedlot receiving), number of animals receiving each treatment, treatment means, and a measure of effect with the ability to calculate the standard deviation of treatment group for performance data or an odds ratio for health outcomes.

### Data extraction & classification

Based on study objectives, the following data were extracted from included studies: phase where supplementation occurred, cattle risk classification, additional trace mineral supplementation, length of study, breed of animals utilized, animal diet, ADG, and overall morbidity outcomes. Data were extracted as treatment means, standard error of the means, standard deviations, *P*-values, and odds ratios.

Data were further classified based on phase receiving supplementation, risk classification, ITM product, concurrent use of additional trace mineral products, length of study, and breed of animals utilized. Phase receiving supplementation was separated into preconditioning or feedlot receiving, defined by administering an ITM product at the beginning of the preconditioning phase or on arrival to a feedlot facility. Risk classification was separated into high- and low- risk, with “high-risk” studies utilizing animals with no known previous vaccination and management history or sourced from an auction facility (Groves 2020). Studies were classified as “low-risk” if animals with known vaccination and management history or sourced from a research facility were used. Studies which utilized additional Cu, Mn, Se, and Zn via oral supplementation methods, such as granular mineral or as a pre-mix within feed, were classified as “YES”, while studies that did not include this additional supplementation were classified as “NO.” Length of study was classified as “short” if researchers investigated effects of a singular ITM dose for less than or equal to 30 days, “moderate” if study duration was between 30 and 60 days, and “extended” if the study duration was greater than 60 days long. Length of study classifications were determined based on 1) certified industry preconditioning program length requirements (Texas Cooperative Extension) and 2) average receiving period durations reported by surveyed feedlot nutritionists (Samuelson et al. 2016). Animal breed classification was defined as *B. taurus* (ie, Angus), *B. indicus* (ie, Nellore), or crossbred (ie, Brangus) as reported by authors of the study. Due to only one study including the usage of a metaphylactic antibiotic injection during at-arrival processing for disease mitigation, further analysis was limited and therefore excluded from this analysis.

### Statistical analysis

Meta-analyses on variables of interest outlined in the study objectives were conducted utilizing the metacont and metabin functions for average daily gain and overall morbidity outcomes, respectively, in the meta package of R (v. 4.3.2; R. Foundation of Statistical Computing, Vienna Austria). Egger’s regression test of funnel plot asymmetry (Egger et al. 1997) was utilized to analyze publication bias across studies via the metabias function within the meta package of R (v. 4.3.2; R. Foundation of Statistical Computing, Vienna Austria).

For this analysis, a random-effects model was utilized which included effect size, I^2^ statistics, and τ^2^ statistics for the analysis of variance. Effect size was calculated based on the mean difference between treatments and was standardized utilizing the inverse of its variance and utilized to weigh studies within this analysis (Lean et al. 2009). A χ^2^ test of heterogeneity was utilized to analyzed variances between studies and was calculated via an I^2^ statistic (Egger and Smith 2001). Heterogeneity was then classified based on the I^2^ statistic with 0% = no heterogeneity, 25% = low, 50% = moderate, and >70% = high heterogeneity (Higgins et al. 2003). Additionally, τ^2^ statistics were utilized to further analyze variance between studies within the random effects model (Higgins and Green 2011).

Significance was set at *P ≤* 0.05 and tendencies were determined if *P >* 0.05 and ≤0.10.

## Results & discussion

This current analysis focuses on the effects of a singular-dose injectable trace mineral solution containing Cu, Mg, and Zn chelated to Ethylenediaminetetraacetic acid (**EDTA**) and Se as sodium selenite on the performance and health of cattle during high-stress periods of time, such as preconditioning and feedlot receiving. Studies included in this meta-analysis are described in Table 1, including subclassifications, with specific details relating to number of experimental units and at-arrival processing procedures outlined in Tables 2 and 3, respectively. It should be noted that the current work utilized studies conducted at universities, which may not accurately large-scale commercial trials and results should be interpreted as such. A table describing initial and final body weight, and dry matter (**DM**) intake is provided in supplementary material 1.

**Table 2.** A summation of studies investigating the effects of injectable trace mineral products on animal performance and health in beef cattle including the number of pens within each treatment group, number of animals within each treatment group, and animal sex.

Study	ITM Pens^a^ (n)	CON Pens^a^ (n)	ITM head (n)	CON head (n)	Animal Sex^b^
Arthington et al. (2014) (Exp. 2)*	12	12	12*	12*	Heifer
Caramalac et al. (2021)	6	6	29	29	Heifer
Clark et al. (2006)	9	10	94	96	Steer
da Silva Zornitta et al. (2021)	**	**	15	15	Bull, Heifer
Genther-Schroeder & Hansen (2015) (Study 1)*	2	2	24*	24*	Steer
Genther-Schroeder & Hansen (2015) (Study 2)*	2	2	24*	24*	Steer
Grossi et al. (2025)*	13	14	283*	293*	Bull
Niedermayer et al. (2017*)	7	7	83*	83*	Steer
Rauch et al. (2018) (Study 1)	6	6	34	34	Steer
Rauch et al. (2018) (Study 2)	6	6	34	34	Steer
Richeson and Kegley (2011) (Study 1)	5	5	30	30	Heifer
Richeson and Kegley (2011) (Study 2)	5	5	30	30	Heifer
Roberts et al. (2016)	8	8	64	64	Steer
Vedovatto et al. (2019)*	**	**	79*	80*	N/A
Vedovatto et al. (2024) (Study 1)*	**	**	43*	43*	Bull, Heifer
Vedovatto et al. (2024) (Study 2)*	**	**	25*	25*	Bull

a Pen was utilized at experimental unit for all analyses within the present study, except those denoted by an asterisk (*) due to animal being utilized as experimental unit within the study. Studies denoted by a double asterisk (**) reported animals being held within a singular pasture instead of individual pens.

b Defined by sex of animal at initiation of experiment.

**Table 3.** Summary of studies investigating the effects of injectable trace mineral products on animal performance and health in beef cattle detailing receiving protocols including vaccinations, administration of anthelmintic products, usage of hormonal implants, usage of metaphylactic antibiotics, castration, and dehorning.

Study Name	Vaccination^a^	Anthelmintic^b^	Implant^c^	Metaphylactic Antibiotic^d^ ^,^ ^e^	Castration^f^	Dehorn^g^
Arthington et al. (2014) (Exp. 2)	N	N	N	N	N	N
Caramalac et al. (2021)	N	N	N	N	N	N
Clark et al. (2006)	Y	Y	N	N	N	N
da Silva Zornitta et al. (2021)	N	N	N	N	N	N
Genther-Schroeder & Hansen (2015) (Study 1)	Y	Y	N	N	N	N
Genther-Schroeder & Hansen (2015) (Study 2)	Y	Y	N	N	N	N
Grossi et al. (2025)	Y	N	N	N	N	N
Niedermayer et al. (2017)	Y	Y	N	N	N	N
Rauch et al. (2018) (Study 1)	Y	Y	Y	N	Y	N
Rauch et al. (2018) (Study 2)	Y	Y	Y	N	Y	N
Richeson and Kegley (2011) (Study 1)	Y	Y	N	N	N	Y
Richeson and Kegley (2011) (Study 2)	Y	Y	N	N	N	Y
Roberts et al. (2016)	Y	Y	Y	Y	Y	N
Vedovatto et al. (2019)	N	Y	N	N	N	N
Vedovatto et al. (2024) (Study 1)	Y	N	N	N	N	N
Vedovatto et al. (2024) (Study 2)	Y	N	N	N	N	N

a Y = Animals were vaccinated upon study initiation for at least BRD complex; N = Animals were not vaccinated upon study initiation; N/A = No information regarding initial processing was available.

b Y = Animals were administered an anthelmintic product upon study initiation; N = Animals were not administered an anthelmintic product upon study initiation; N/A = No information regarding initial processing was available.

c Y = Animals were administered a hormonal implant upon study initiation; N = Animals were not administered a hormonal implant upon study initiation; N/A = No information regarding initial processing was available.

d Y = Animals were administered metaphylactic antibiotics upon study initiation; N = Animals were not administered metaphylactic antibiotics upon study initiation; N/A = No information regarding initial processing was available.

e All metaphylactic antibiotics utilized were injectable.

f Bulls were castrated upon arrival via banding method.

g Y = Animals which required dehorning were dehorned upon study initiation; N = Animals were not dehorned upon study initiation; N/A = No information regarding initial processing was available.

**Fig. 1.**
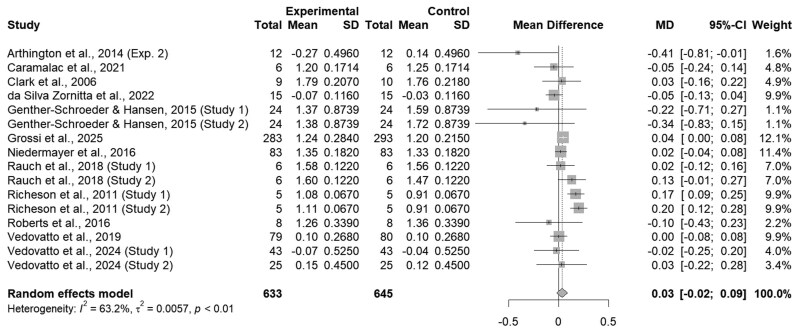
Forest Plot comparing the overall effects of an ITM solution (experimental) to a saline solution (control) on beef cattle ADG during preconditioning or feedlot receiving phases with the treatment mean, standard deviation, mean difference, and study weight described.

When all studies were analyzed together, the administration of an ITM did not impact cattle ADG (*P = *0.21; Fig. 1), however, moderate heterogeneity was present (I^2^ = 63.2%; τ^2^ < 0.01 *P <*0.01; Fig. 1). The lack of effect for ADG with the administration of an ITM solution is unexpected due to role of Cu, Mn, Se, and Zn within protein synthesis and metabolism (Suttle 2021). Stressors associated with preconditioning and feedlot receiving, such as transportation, comingling, and dietary changes, have the potential to decrease initial feed intake and subsequently decrease growth performance (Cooke 2017; Marques et al. 2012; Loerch and Fluharty 2000). More specifically, feed bunk acclimation must be established to ensure effective supplementation through oral methods to maintain adequate trace mineral status. However, feed bunk acclimation may take up to 21–28 days, affecting oral supplementation methods (Felix 2023), whereas ITM may provide a strategy to circumvent decreased feed intake and increase cattle trace mineral status. However, no difference was detected (*P = *0.94). for DM intake for cattle administered an ITM or not. For example, Pogge et al. (2012) demonstrated an increase in plasma Mn and Zn until 12-hours post-injection and plasma Se for 24-hours post-injection. However, these values were comparable to a saline injection after 12-, 12-, and 24- hours for Mn, Zn, and Se, respectively, indicating a decrease in circulating trace mineral concentrations shortly after injection time.

**Fig. 2.**
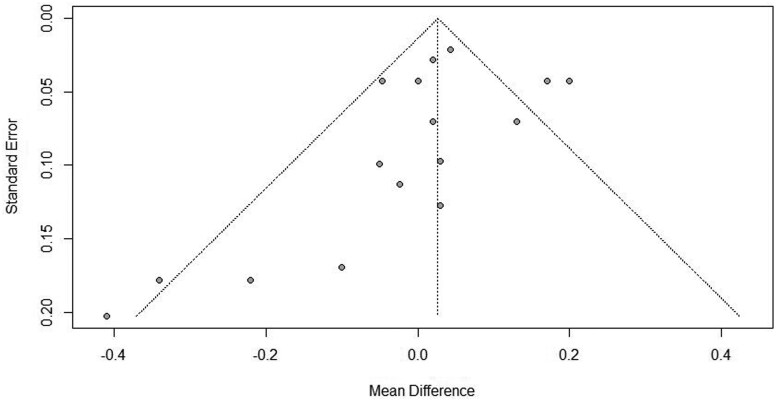
Egger’s regression test for funnel plot asymmetry for ADG investigating publication bias of articles included within the present analysis.

An Eggar’s regression test for funnel plot asymmetry was not significant for ADG (*P = *0.18; Fig. 2), indicating a lack of publication bias for this analysis. These results indicate that published manuscripts surrounding the effects of ITM products on ADG were representative of all studies conducted and indicative of true effects of such products.

**Fig. 3.**
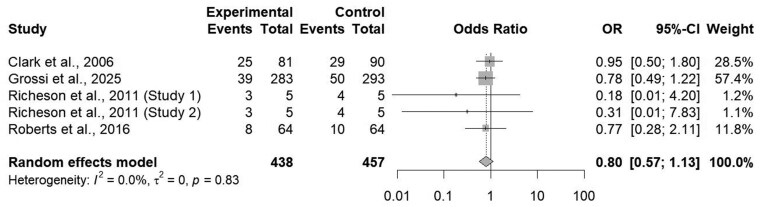
Forest Plot comparing the effects of an ITM solution (experimental) to a saline solution (control) on overall cattle morbidity within preconditioning and feedlot receiving with the treatment mean, standard deviation, mean difference, and study weight described.

Additionally, the administration of an ITM product did not affect overall morbidity outcomes within the preconditioning and feedlot receiving phases (*P = *0.20; Fig. 3) with heterogeneity found to be null (I^2^ = 0.0%; τ^2^ = 0.00; *P = *0.83; Fig. 3). An Eggar’s regression test for funnel plot asymmetry for morbidity could not be conducted due to less than 10 studies providing extractable morbidity data for this analysis, severely underpowering an assessment on publication bias. It should be noted this analysis was conducted utilizing overall morbidity defined as animals exhibiting clinical signs of illness requiring pharmaceutical intervention or removal from treatment pen as reported by study authors, not morbidity related to specific pathogens or diseases (ie, BRD, interdigital dermatitis, etc).

This lack of effect on overall morbidity seen is unexpected due to the roles of ITM supplemented trace minerals, such as Cu, Mn, Se, and Zn, have within immune system function and stability. For example, Cu functions within the acute phase protein ceruloplasmin to aid in antioxidant facilitation during periods of increased stress to prevent superoxide radical damage to healthy cells (Gropper et al. 2018). Similar to Cu, Mn functions within superoxide dismutase to prevent reactive oxygen species damage from phagocytotic cells (Palomares 2022), however, evidence of its role outside of superoxide dismutase is limited. Selenium plays a crucial role within the innate immune system through neutrophil migration and inflammation, with a deficiency shown to have decreased neutrophil migration, reduced glutathione peroxidase function, and impaired B-cell activity (Boyne and Arthur 1981; Maddox et al. 1999). Zinc, which plays a role in over 300 enzymes within the body (Gropper et al. 2018), functions within the innate and acquired immune system in a variety of ways. Its involvement in protein synthesis and gene expression is essential during times of heightened stress, supporting cytokine production, lymphocyte development, and the repair of mucosal cells (Palomares 2022).

Nevertheless, several studies (*n* = 5) included additional immunological response data such as antibody titers for porcine red blood cells (**PRBC**) or bovine viral diarrhea virus (**BVDV**) and blood leukocyte profiles in response to ITM administration. Bittar et al. (2018) reported administration of an ITM product improved immune response to specific bacterial pathogens related to BRD while Hoyos-Jaramillo et al. (2022) demonstrated cattle receiving ITM had reduced upper respiratory tract inflammation following a BVDV-2 and bovine herpes virus-1 (**BHV-1**) challenge subsequent to intranasal vaccination compared to control cohorts. However, infections with BVDV and BHV-1 frequently occur in the absence of overt clinical signs but serve as a pathway for additional comorbidities, potentially altering health outcome data presented by authors of studies included in this analysis through the underreporting of such morbidities (Jones 2003; Campbell 2004; Jones and Chowdhury 2010). These ITM products may improve immune system response against pathogens relating to BRD, however, further research exploring the effectiveness of ITM products at reducing overall morbidity during the preconditioning and feedlot receiving phases is warranted.

Due to the lack of studies utilizing ITM products during preconditioning and feedlot receiving that provided accessible morbidity data, further subclass analysis on morbidity outcomes was underpowered and statistically limited to this initial overall analysis. Therefore, the remainder of this manuscript will be centered on the effects of ITM administration on cattle growth performance outcomes.

### Risk classification

**Fig. 4.**
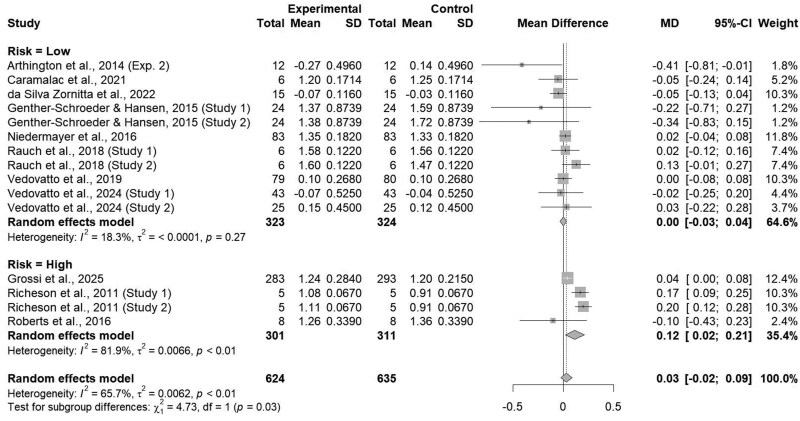
Forest Plot comparing the effects of an ITM solution (experimental) to a saline solution (control) on average daily gain by animal risk classification, either high- or low-risk with the treatment mean, standard deviation, mean difference, and study weight described.

Individual studies included within this analysis were further classified based on animal risk status. Studies were classified as high-risk (*n* = 4) if no known previous management or vaccination history was known to researchers prior to the study and low-risk (*n* = 11) if previous management and/or vaccination history was known prior to the study. Average daily gain differed between risk classification, either high- or low-risk (*P = *0.03; Fig. 4). The administration of an ITM product increased ADG by 0.12 kg/d (*P = *0.02) in high-risk cattle, however the same effect was not seen within the low-risk cattle subclass (*P = *0.93). Differences for ADG between risk classes could be attributed to differences in liver mineral status and subsequent utilization potential. High-risk cattle are usually procured from auction facilities or sale barns and often exhibit considerable variability in initial liver mineral concentrations, which may predispose animals to a higher risk of trace mineral deficiencies (Stewart et al. 2010). However, few studies in this subclass analysis (*n* = 5) evaluated at arrival liver mineral status in low-risk cattle, with all studies reporting adequate liver trace mineral status for all trace minerals supplemented within an ITM injection. With no studies present within this meta-analysis utilizing high-risk cattle reporting at-arrival liver mineral status, limiting further analysis regarding the effects of an ITM product on liver mineral status in high-risk cattle. Moreover, Richeson and Kegley (2011) reported no difference between plasma Cu and Zn when utilizing two ITM products and a saline injection in high-risk cattle. However, it should be noted that authors of this study only reported plasma Cu and Zn concentrations for d0 and d28 of the study. With ITM products intended for rapid supplementation, differences within plasma concentrations of Cu and Zn could have been overlooked due to the lengthy interval between sample collections.

On the other hand, heterogeneity was found to be null within the low-risk subclass (I^2^ = 18.3%; τ^2^ < 0.01; *P = *0.27) but high within the high-risk category (I^2^ = 81.9%; τ^2^ < 0.01; *P <*0.01). The high heterogeneity found within the subclass of studies utilizing high-risk cattle demonstrates a lack of cohesivity across research, possibly due to the diverse stressors characteristic of the preconditioning and feedlot receiving period. However, it should be noted that only four studies from three manuscripts were utilized within the high-risk subclass, potentially affecting results due to a limited amount of data present. Heterogeneity was found to be null within the low-risk subclass, demonstrating a lack of variability across studies. The lack of effect on ADG coupled with null heterogeneity seen within this subclass could potentially be attributed to management strategies implemented prior to studies, such as trace mineral supplementation, proper vaccination, and low stress weaning methods. Prior trace mineral supplementation could ensure adequate mineral status, decreasing the performance effects of additional supplementation through an ITM solution (Arthington and Ranches 2021). Moreover, proper vaccination protocols could decrease the initial physiological overload due to pathogen introduction during preconditioning or feedlot receiving, potentially affecting ADG through increased feed intake (Lippolis et al. 2016). Sowell et al. (1999) demonstrated a decrease in feeding and water consumption events within high-risk, morbid steers during the first 4 days of feedlot receiving compared to healthy steers, further supporting a need for proper health management to maximize feedlot productivity. Moreover, lower-stress weaning strategies, such as weaning on farm via fence-line or nose-flap methods prior to shipment or creep feeding, prior to study implementation could allow animals to acclimate to new conditions during preconditioning and feedlot receiving. Arthington et al. (2008) demonstrated that cattle which were creep feed and weaned prior to shipment had an increased ADG during a 29-day feedlot receiving period compared to control calves which were weaned at time of shipping, potentially due to the prior acclimation to feedlot receiving conditions. Additionally, researchers highlighted the decrease in acute phase protein response in cattle which were early weaned and allowed to remain on farm until shipping compared to control calves, which has been shown to be negatively correlated with receiving ADG (Arthington et al. 2005, 2008). These combined management strategies prior to preconditioning and feedlot receiving could have decreased the effectiveness of an ITM solution due to low-risk animals being physiologically stable at time of administration, in which additional supplementation was not necessary to improve animal performance.

### Production phase

**Fig. 5.**
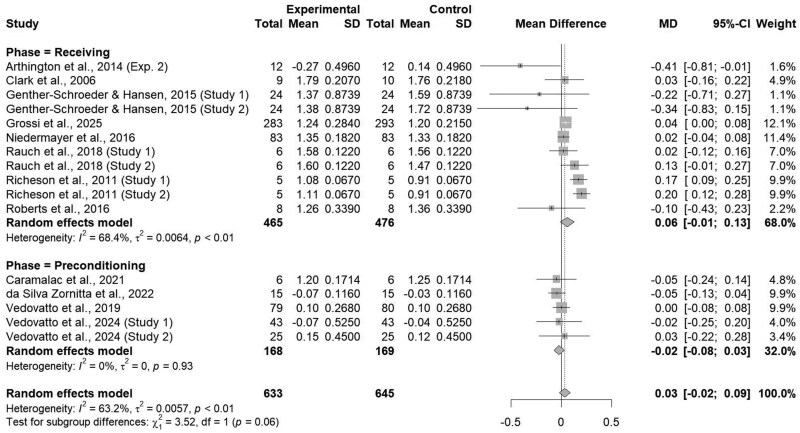
Forest Plot comparing the effects of an ITM solution (experimental) to a saline solution (control) on average daily gain production phase, either preconditioning or feedlot receiving with the treatment mean, standard deviation, mean difference, and study weight described.

Individual studies were classified based on the production phase in which the ITM product was utilized, either preconditioning or feedlot receiving. Average daily gain tended to differ (*P = *0.06; Fig. 5) between preconditioning and feedlot receiving, accordingly, the usage of an ITM product did not affect ADG within the preconditioning phase (*P = *0.39). However, the usage of an ITM product during feedlot receiving tended (*P = *0.09) to increase ADG by 0.06 kg/d.

Although both preconditioning and feedlot receiving are recognized as stressful periods, the nature and intensity of the stressors encountered during each phase differ (Cooke 2017), which may result in variable responses ITM administration. Arthington et al. (2014) demonstrated an increase in plasma ceruloplasmin, haptoglobin, and acid soluble protein for an extended period of time in calves administered an ITM injection compared to calves administered saline at arrival to a feedlot receiving. These results indicate an increase in physiological stress, potentially due to an inflammatory response to the chelating agent of EDTA. Researchers also reported an increase in acute phase proteins, specifically ceruloplasmin and haptoglobin, which have been identified as being negatively correlated ADG during feedlot receiving and positively correlated with feedlot morbidity (Berry et al. 2004; Arthington et al. 2005). However, González-Cuevas et al. (2011) demonstrated EDTA can possess anti-inflammatory actions within rodents challenged with a carcinogenic substance, further raising questions surrounding inflammatory processes and EDTA usage as a chelating agent within ITM solutions. While intended to improve physiological responses to stress through additional supplementation of Cu, Mn, Se, and Zn, the subsequent inflammatory response caused may decrease the efficacy of such product through the promotion of the acute phase protein response.

Furthermore, the heterogeneity between studies for ADG were null within the preconditioning subclass (I^2^ = 0.0%; τ^2^ = 0.00; *P = *0.93) but moderate within the feedlot receiving class (I^2^ = 68.4%; τ^2^ < 0.01; *P <*0.01). These results indicate cohesivity within the literature surrounding the effects on ADG within the preconditioning subclass. However, the moderate heterogeneity found within the feedlot receiving subclass could potentially be attributed to a wide variety of animal differences not seen within the preconditioning subclass. For example, within the preconditioning subclass all studies utilized low-risk cattle. However, within the feedlot receiving subclass, 54.5% (6/11) of studies utilized low-risk cattle, 36.4% (4/11) studies utilized high-risk cattle, and one study utilized a combination of low- and high-risk cattle. As previously mentioned, an ITM product could be more beneficial within high-risk cattle when compared to low-risk cattle, potentially contributing to the moderate heterogeneity within this subclass. However, studies within the preconditioning subclass utilized purebred cattle while the majority of studies within the feedlot receiving subclass utilized crossbred cattle. Specific breed differences could also contribute to this moderate heterogeneity present, and these are discussed in detail further in this analysis.

### Additional trace mineral supplementation

**Fig. 6.**
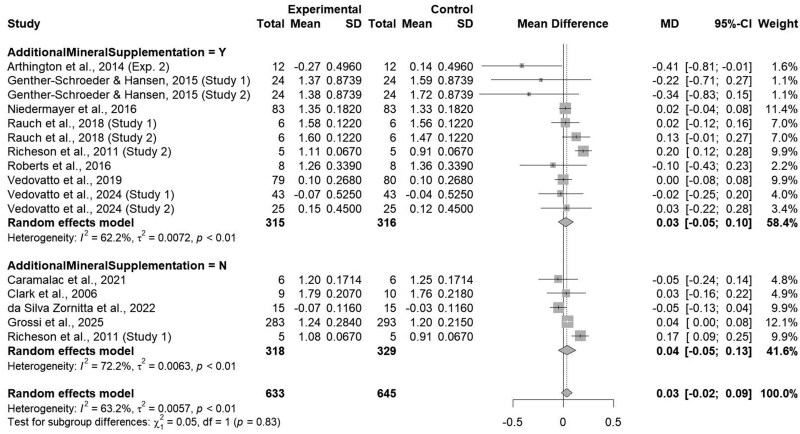
Forest Plot comparing the effects of an ITM solution (experimental) to a saline solution (control) on average daily gain by usage of additional trace mineral supplementation with the treatment mean, standard deviation, mean difference, and study weight described.

Average daily gain did not differ based on the inclusion of additional oral trace mineral supplementation post-injection with an ITM product (*P = *0.83; Fig. 6), accordingly, ADG was not affected within studies including additional trace mineral supplementation (*P = *0.49) or studies that did not include additional trace mineral supplementation (*P = *0.37). Additionally, authors who utilized additional trace mineral supplementation (*n* = 8) reported a variety of oral supplementation sources, either organic or inorganic. Previous research conducted by our group (McKnight et al. 2025) reported trace mineral supplementation via organic sources may increase cattle ADG when compared to inorganic sources during preconditioning and feedlot receiving. With the variability in mineral sources present within studies that utilized additional trace mineral, the potential conflicting effects of oral organic and inorganic sources could have altered ADG results. However, manufacturer label recommendations express additional trace mineral supplementation post-ITM administration carries potential toxicity risks due to ITM products immediately entering circulation, specifically for Cu and Se due to the thin margin between animal requirements and toxicity levels. The recommended concentration of Cu within beef cattle rations is 10 mg Cu/kg DM, however requirements may range from 4 to ≥15 mg Cu/kg DM due to dietary S or Mo with potential toxicity at 40 mg Cu/kg DM (NASEM 2016). Likewise, the recommended Se concentration within beef rations is 0.1 mg Se/kg DM with potential toxicity at 2 mg Se/kg DM (NASEM 2016). Due to the thin margin between animal requirements and toxicity, supplementing trace minerals, specifically Cu and Se, to animals orally in addition to an ITM solution could lead to negative effects, including decreased performance or mortality (NASEM 2016). While no study reported this complication post-injection, the risk of toxicity remains viable and should be considered when utilizing such products. However, this toxicity is not likely during periods of decreased feed intake, such as during preconditioning or feedlot receiving. Reduced dietary consumption inherently limits the total amount of trace minerals ingested, making it less probable that intake will exceed toxic thresholds.

Additionally, heterogeneity was found to be moderate within the “YES” subclass (I^2^ = 62.2%; τ^2^ = 0.01; *P <*0.01) and “NO” subclass (I^2^ = 72.2%; τ^2^ < 0.01; *P <*0.01) for additional trace mineral supplementation. Heterogeneity found within the “YES” subclass can be attributed to varying trace mineral sources, either organic or inorganic, as previously mentioned. However, few studies did not include additional trace mineral supplementation post-injection (*n* = 3), potentially altering heterogeneity within this subclass and indicating a need for additional investigation into the usage of an ITM product with no subsequent oral supplementation to further quantify the lone effects of an ITM product.

### Length of study

**Fig. 7.**
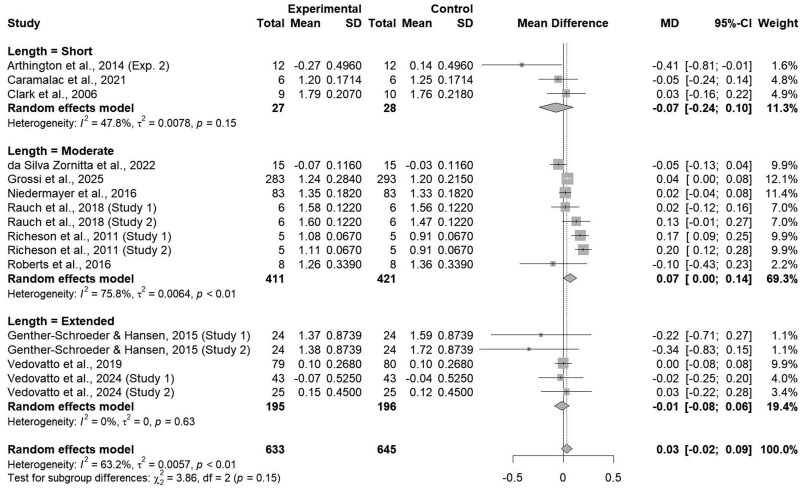
Forest Plot comparing the effects of an ITM solution (experimental) to a saline solution (control) on average daily gain by study duration, either short, moderate or extended with the treatment mean, standard deviation, mean difference, and study weight described.

Average daily gain tended to differ (*P = *0.14; Fig. 7) across study length durations, accordingly, the usage of an ITM product did not influence ADG in shorter study durations (≤ 30 days; *P = *0.41) or extended study durations (≥ 60 days; *P = *0.74) while ADG was increased by 0.07 kg/d (*P = *0.04) within studies in the moderate study duration subclass. The lack of effect on cattle ADG during shorter study durations was contrary to initial expectations due to the quick absorption and subsequent circulation of ITM products. Pogge et al. (2012) demonstrated administration of a single-dose ITM results in prompt increases circulating trace mineral concentrations in cattle with already adequate liver trace mineral status. More specifically, these authors reported plasma Mn and Zn increased significantly 8 and 12 hours post ITM administration, and plasma Se increased significantly 8, 10, and 24 hours post ITM administration (Pogge et al. 2012). Likewise, Genther and Hansen (2014) demonstrated an ITM could improve liver Cu and Se concentrations during a repletion period through at least 29 days post-injection in auction-derived steers fed a common finishing diet after an 84-day trace mineral depletion period. However, little research is available surrounding the exact duration of action of ITM products, specifically circulation and utilization times, for extended periods of time. Arthington and Ranches (2021) described the high variability regarding the ability of individual animals to effectively store and utilize micronutrients, especially during periods of trace mineral deficiencies or stress.

Heterogeneity was found to be null within the short (I^2^ = 47.8%; τ^2^ < 0.01; *P = *0.15) and extended (I^2^ = 0.0%; τ^2^ < 0.01; *P = *0.63) subclasses. However, high heterogeneity was present (I^2^ = 75.8%; τ^2^ < 0.01; *P <*0.01) within the moderate subclass. The high heterogeneity in studies analyzing the effects of ITM on ADG between 30 and 60 days can be attributed to differences across studies within this subclass, potentially due to this subclass covering both typical preconditioning lengths and extended feedlot receiving phases. The Value-Added Calf (**VAC**) program (2005) through Texas A&M Cooperative Extension program requires producers to precondition animals for at least 45 days prior to shipment, however, many studies within this subclass (*n* = 7) utilized an extended receiving period ranging from 42 to 56 days. Additionally, diets fed to animals within this subclass ranged from forage-based to corn-based concentrates, potentially altering ADG and heterogeneity due to animals fed corn-based diets having an increased ADG compared to those on forage-based diets (Duckett et al. 2013). The null heterogeneity within the short and extended study duration subclasses consistently demonstrates a lack of effect on ADG across studies less than or equal to 30 days or greater than 60 days in length. The combined lack of effect coupled with null heterogeneity within this subclass, which is not seen within the moderate subclass, could indicate a time period of between 30 and 60 days in which ITM could be most effective at increasing ADG.

### Animal breed

**Fig. 8.**
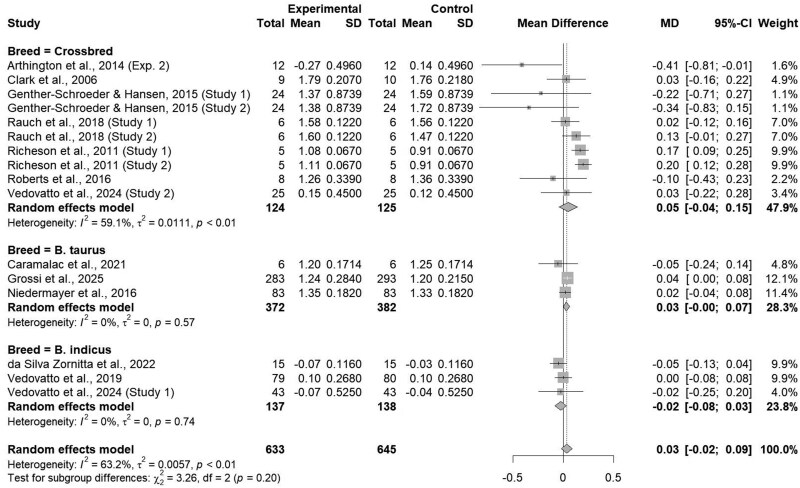
Forest Plot comparing the effects of an ITM solution (experimental) to a saline solution (control) on average daily gain by animal breed, either *B. taurus*, *B. indicus*, or crossbred with the treatment mean, standard deviation, mean difference, and study weight described.

Studies utilized within this subclass analysis were classified based by breed utilized within the study as reported by authors, either *B. indicus*, *B. taurus*, or crossbred. Average daily gain was similar between breed classifications (*P = *0.20; Fig. 8). Injectable trace mineral usage did not affect ADG within *B. indicus* (*P = *0.42). However, ITM usage increased ADG (*P = *0.05) by 0.03 kg/d within the *B. taurus* subclass and increased ADG (*P = *0.02) by 0.09 kg/d within the crossbred subclass. Differences within subspecies of cattle could be a plausible explanation for the lack of effect within *B. indicus*. Ranches et al. (2021) demonstrated Brahman cattle had a greater liver Cu concentration when compared to Angus cattle during a 90-day restriction phase and a 60-day supplementation phase with a tendency for Angus cattle to have greater liver Se during the restriction phase. Additionally, Brahman cattle had greater concentrations of circulating Cu during the restriction and supplementation phases when compared to Angus cattle, while Angus cattle had greater concentrations of circulating Se during both phases. On the other hand, Pogge et al. (2012) demonstrated a difference in plasma Cu, Zn, and Se with Angus cattle having greater circulating trace mineral levels than Simmental cattle, indicating a potential breed influence within the *B. taurus* subclass on trace mineral status. These results indicate that different subspecies of cattle may be more efficient at maintaining adequate statuses of varying trace minerals, potentially due to evolutionary traits stemming from their environmental origins where forage availability and nutrient quality differ (Cooke et al. 2020). However, the exact metabolic mechanisms behind this difference are not thoroughly understood.

Furthermore, heterogeneity was found to be null within the *B. indicus* (I^2^ = 0.0%; τ^2^ = 0.00; *P = *0.19) and *B. taurus* (I^2^ = 0.00%; τ^2^ = 0.00; *P = *0.57) subclasses. However, heterogeneity had a tendency to be low (I^2^ = 46.5%; τ^2^ < 0.01; *P = *0.06) within the crossbred subclass. This low heterogeneity could be attributed to the breed differences found within *B. indicus* and *B. taurus* cattle combined to create hybrid vigor differing in each individual animal, leading to differing effects between studies.

## Conclusions

Through the integration of diverse datasets across multiple sources, this meta-analysis generated fresh insights into both the effectiveness and implications of ITM usage during preconditioning and feedlot receiving on cattle growth performance and health outcomes. This analysis suggests that the implementation of ITM products during these specific phases does not have an effect on both growth performance and morbidity. While this analysis aided in identifying a potential association between the increase in ADG within high-risk cattle, study durations, and cattle breed, further research is warranted to clarify underlying physiological mechanisms of such products within preconditioning and feedlot receiving.

## Funding

This publication is a contribution of the Mississippi Agricultural and Forestry Experiment Station, Mississippi State University. The authors acknowledge the USDA-National Institute of Food and Agriculture Multistate Project S1093—Management systems for beef cattle reared in subtropical and tropical environments.

## Supplementary Data

Supplementary data is available at *Translational Animal Science* online.

## CRediT authorship contribution statement

**M. G. McKnight:** Conceptualization, Methodology, Investigation, Formal analysis, Writing—original draft, Visualization. **K. M. Harvey:** Conceptualization, Methodology, Investigation, Formal analysis, Writing—original draft, Visualization, Supervision. **W. I. Jumper:** Conceptualization, Methodology, Writing—reviewing & editing, Supervision. **J. Ranches:** Methodology, Investigation, Writing—reviewing & editing. **B. B. Karisch:** Methodology, Investigation, Writing—reviewing & editing.

## Disclosures

No authors have no conflicts of interest to report.

## Supplementary Material

txaf162_Supplementary_Data

## Abbreviations:

AI: artificial insemination
ADG: average daily gain
BCS: body condition score
BHV: bovine herpes virus
BRD: bovine respiratory disease
BVDV: bovine viral diarrhea virus
DM: dry matter
EDTA: Ethylenediaminetetraacetic acid
ITM: Injectable trace minerals
PRBC: porcine red blood cells
