# Supplementary material for: The effects of injectable trace mineral solutions on beef cattle performance and health during preconditioning and feedlot receiving: a systematic review and meta-analysis
Source: Transl Anim Sci. 2025 Dec 13;10:txaf162. doi: 10.1093/tas/txaf162 (PMC12804072; doi:10.1093/tas/txaf162)
Supplement: txaf162_Supplementary_Data [file txaf162_supplementary_data.docx]

|  | **Initial BW (kg)** | | | **Final BW (kg)** | | | **DMI (kg/d)** | | |
| --- | --- | --- | --- | --- | --- | --- | --- | --- | --- |
| **Study** | **ITM** | **CON** | **SE** | **ITM** | **CON** | **SE** | **ITM** | **CON** | **SE** |
| Arthington et al., 2014 (Exp. 2) | 220 | 241 | 3.7 | 216 | 221 | 3.7 | -- | -- | -- |
| Caramalac et al., 2021 | 236 | 237 | 7.0 | 270 | 271 | 2.0 | 5.51 | 5.79 | 0.14 |
| Clark et al., 2006 | 266 | 266 | 27 | -- | -- | -- | -- | -- | -- |
| da Silva Zornitta et al., 2022 | 176.78 | 176.66 | 1.24 | 171.64 | 174.50 | 1.24 | -- | -- | -- |
| Genther-Schroeder & Hansen, 2015a | 298 | 297 | 3.5 | 273 | 273 | 2.9 | 6.41 | 6.89 | 0.159 |
| Genther-Schroeder & Hansen, 2015b | 299 | 298 | 3.5 | 277 | 278 | 2.9 | 6.82 | 6.85 | 0.159 |
| Grossi et al., 2025 | 389.67 | 383.33 | 1.20, 1.83 | 448.64 | 446.79 | 0.65, 0.66 | 8.45 | 8.77 | 0.07 |
| Niedermayer et al., 2016 | 358 | 358 | 1.5 | 434 | 433 | 1.5 | 8.60 | 8.91 | 0.156 |
| Rauch et al., 2018a | 196 | 196 | 2.0 | 263 | 258 | 2.4 | 5.4 | 5.3 | 0.15 |
| Rauch et al., 2018b | 200 | 199 | 2.0 | 262 | 265 | 2.4 | 5.4 | 4.8 | 0.15 |
| Richeson et al., 2011a | 198.7 | 199 | 6.42 | 258.4 | 248.9 | 7.02 | 5.66 | 5.23 | 0.13 |
| Richeson et al., 2011b | 199.2 | 199 | 6.42 | 260.1 | 248.9 | 7.02 | 5.57 | 5.23 | 0.13 |
| Roberts et al., 2016 | 275 | 275 | 9.54 | 328 | 332 | 10.56 | 7.02 | 6.98 | 0.27 |
| Vedovatto et al., 2019 | 213 | 213 | 32 | 234 | 233 | 0.85 | -- | -- | -- |
| Vedovatto et al., 2024a | 198 | 198 | 0.94 | 196 | 195 | 0.94 | -- | -- | -- |
| Vedovatto et al., 2024b | 264 | 264 | 3.29 | 291 | 293 | 3.29 | -- | -- | -- |

**Supplemental Table 1.** A summation of studies detailing initial body weight, final body weight, and dry matter intake (DMI) as reprted by study authors for each treatment group (ITM and CON).
